# Preparation and Identification of Optimal Synthesis Conditions for a Novel Alkaline Anion-Exchange Membrane

**DOI:** 10.3390/polym10080913

**Published:** 2018-08-13

**Authors:** Aitor Marcos-Madrazo, Clara Casado-Coterillo, Leticia García-Cruz, Jesús Iniesta, Laura Simonelli, Víctor Sebastián, María del Mar Encabo-Berzosa, Manuel Arruebo, Ángel Irabien

**Affiliations:** 1 Department of Chemical and Biomolecular Engineering, Universidad de Cantabria, 39005 Santander, Spain; marcosma@unican.es (A.M.-M.); irabienj@unican.es (Á.I.); 2 Department of Physical Chemistry and Institute of Electrochemistry, University of Alicante, 03080 Alicante, Spain; leticia.garcia@ua.es (L.G.-C.); jesus.iniesta@ua.es (J.I.); 3 CELLS—ALBA Synchrotron Radiation Facility, Carrer de la Llum 2-26, 08290 Cerdanyola del Vallès, Barcelona, Spain; lsimonelli@cells.es; 4 Department of Chemical and Environmental Engineering, Instituto de Nanociencia de Aragón, Universidad de Zaragoza, 50018 Zaragoza, Spain; victorse@unizar.es (V.S.); mmar@unizar.es (M.d.M.E.-B.); arruebom@unizar.es (M.A.); 5 Networking Research Center on Bioengineering, Biomaterials and Nanomedicine, CIBER-BBN, 28029 Madrid, Spain

**Keywords:** ion exchange membranes, renewable and economic polymers, copper based fillers, ANOVA, regression analysis, water vapor permeability (WVP), anion conductivity, X-ray absorption near edge structure (XANES)

## Abstract

The physicochemical and mechanical properties of new alkaline anion-exchange membranes (AAEMs) based on chitosan (CS) and poly(vinyl alcohol) (PVA) polymers doped with unsupported copper nanoparticles (NPs) and copper exchanged over different porous materials were investigated regarding ion-exchange capacity (IEC), OH^−^ conductivity, water uptake (WU), water vapor permeability (WVP), and thermal and mechanical resistance. The influence of the type of filler included in different morphologies and filler loading has been explored using copper exchanged materials such as the layered porous titanosilicate AM-4, layered stannosilicate UZAR-S3, and zeolites Y, MOR, and BEA. Compared to commercially available anion-exchange membranes, the best performing membranes in terms of WU, IEC, OH^−^ conductivity and WVP in this study were those containing 10 wt % of Cu-AM-4 and Cu-UZAR-S3, although 10 wt % Cu-MOR provided better mechanical strength at close values of WVP and anion conductivity. It was also observed that when Cu was exchanged in a porous silicate matrix, its oxidation state was lower than when embedded as unsupported metal NPs. In addition, the statistical analysis of variance determined that the electrochemical properties of the membranes were noticeably affected by both the type and filler loading, and influenced also by the copper oxidation state and content in the membrane, but their hydrophilic properties were more affected by the polymers. The largest significant effects were noticed on the water sorption and transport properties, which gives scope for the design of AAEMs for electrochemical and water treatment applications.

## 1. Introduction

Alkaline anion-exchange membranes (AAEMs) are gaining relevance in the electrochemistry field [1], presenting a wide range of sustainable applications. AAEMs are generally designed to conduct anions preferentially and they are usually prepared from positively charged polyelectrolytes. The major challenges in the development of AAEMs are the improvement in their commonly low OH^−^ conductivity and the required water management to maintain OH^−^ transport from cathode to anode [2,3], whose regulation has been attempted by many procedures such as developing specific flow configurations [4], coating hydrophobic layers on hydrophilic AAEMs [5], ion-exchanging metallic ions in conductive polymers [6], and so on.

Currently, the preparation of AAEMs usually involves the modification of pristine polymers or the direct polymerization of functionalized monomers, where the large amount of organic solvents used during both the reaction and membrane formation processes brings toxicity risks to the environment. Hence, to achieve industrial-scale manufacture, it is important to develop simple, rapid, and environmentally friendly methods for preparing AAEMs [7]. Employing low-cost or renewable polymers, as well as nontoxic inorganic fillers in the synthesis of new membranes is also a way of improving the sustainability of the process. To this end, chitosan (CS) and poly(vinyl) alcohol (PVA) constitute some of the renewable or low-cost alternative in AAEMs for electrochemical processes because its high hydrophilicity and ion exchange capacity, controllable permeability, and consequent energy savings [8,9]. Equimolar blends of CS and PVA were previously proposed as alkaline resistant AAEMs [10], in pervaporation [11,12] and adsorption processes [13].

The swelling by water or any other aqueous media in hydrophilic polymer membranes such as CS:PVA membranes can be further tuned-up by their hybridization with copper nanoparticles (NPs) [14,15]. However, the mechanical stability and reproducible fabrication can be compromised by the interactions of those metal nanoparticles with the ionic charged groups of the polymer chains [15,16]. The incorporation of inorganic moieties in a polymer matrix may increase the mechanical integrity of the membranes [17]. Inorganic moieties can be incorporated in a polymer matrix by: (i) in situ growth of unsupported NPs [16,18]; (ii) introduction of separately prepared or commercial unsupported metal NPs [19], and ion-exchanging the metal cation onto ion-exchangeable materials such as layered titanosilicates [20], or three-dimensional zeolite particles [21].

In this work, we optimized the synthesis of AAEMs prepared from CS:PVA, embedded with inorganic fillers: metal NPs, in the ion-exchangeable nanoporous layered AM-4 titanosilicate and the UZAR-S3 stannosilicate, as well as in different zeolite topologies as Y, MOR, and BEA to improve the membrane properties. The membrane performance was explored in terms of anion-exchange capacity (IEC), hydroxide anion conductivity, water vapor permeability (WVP), and water uptake (WU), and they were compared with those retrieved from a commercial anion exchange membrane.

## 2. Materials and Methods

The polymers employed in this study were chitosan (CS, coarse ground flakes and powder) with molecular weight between 310,000–375,000 Da and 75% deacetylation degree, and poly(vinyl) alcohol (PVA, powder, 99+% hydrolyzed) with molecular weight from 85,000–120,000 Da, both obtained from Sigma-Aldrich, Madrid, Spain, and used as received. Commercial copper nanoparticles (60–80 nm particle size, Sigma-Aldrich, Spain) were used as one of the fillers. The commercial FAA-3 AEM was supplied by Fumatech BWT GmbH (Bietigheim-Bissingen, Germany) and treated in the same way as the prepared membranes for comparison purposes.

### 2.1. Layered Silicates

The reagents used for the layered materials synthesis were: sodium silicate solution (2 wt % SiO_2_, 8 wt % Na_2_O, Merck, Madrid, Spain), titanium(IV) oxide, and tin(II) chloride dihydrate (both from Sigma Aldrich, Spain). Other chemicals were acetic acid (glacial), hydrochloric acid (37%), and sodium hydroxide (pellets), all of them obtained from PANREAC, Barcelona, Spain, and used as received.

The synthesis of layered porous silicates by hydrothermal crystallization was reported elsewhere [22,23]. For the preparation of AM-4 titanosilicate, sodium silicate solution (10.05 g) was mixed with NaOH (1.42 g) and deionized water (6.54 mL). Then, TiO_2_ anatase (0.77 g) was added, rendering a gel with pH 12.1. The gel was seeded on previously synthesized AM-4 (0.08 g) (corresponding to the 0.45 wt % of the mixture) in order to favor the crystallization. After mixing for 1 h, the gel was poured into Teflon-lined autoclaves and heated at 230 °C for 6 h. The typical amount of AM-4 obtained from one synthesis batch was 2 g.

For the UZAR-S3 stannosilicate synthesis [23], the same silicate source as the one used for the AM-4 preparation was employed. The tin source was then added as tin(II) chloride dehydrate (2.41 g) to the mixture, and stirred for 90 min. The gel was introduced in a Teflon-lined autoclave and heated at 230 °C for 96 h. The typical amount of UZAR-S3 collected product was 4 g.

After both syntheses, the solid obtained was washed and filtered three times with 50 mL of deionized water each and dried at 70 °C.

The addition of Cu to the layered silicates was performed by an ion-exchange process to replace de Na^+^ ions with Cu^2+^, as previously reported [20]. The amount of layered material incorporated in the ion-exchange process was 0.5 g. A 10 mM solution of Cu^2+^ was prepared by dissolving Cu(II) nitrate in 50 mL of water. The layered material and the Cu^2+^ solution were mixed in a beaker under continuous stirring. The process was controlled by measuring the pH of the mixture every 10 s until reaching the equilibrium (Figure S1 in the Supporting Information). Although for both layered materials the pH was stabilized almost immediately, the process was maintained for 10 min to assure that the equilibrium was reached. The differences in the final value of the pH after the exchange process with AM-4 and UZAR-S3 are shown in Figure S1 and they might indicate that the ion exchange was more efficient when using the former material.

### 2.2. Zeolites

Fresh and metal-exchanged zeolites were prepared from Na^+^ or NH_4_^+^ zeolites supplied by Zeolyst Int. (Products CBV10A (MOR), CBV100 (zeolite Y), and CP814E (BEA)) with Si/Al ratios equal to 5.1, 13, and 25, respectively, following the work previously reported by Abu-Zied [21].

### 2.3. Membrane Preparation

Membranes were prepared from equimolar blends of CS and PVA dissolved in acidic aqueous media and solution casted as reported elsewhere [10]. Mixed matrix membranes (MMMs) were prepared by adding different inorganic filler loadings from 5 to 15 wt % of the total solid to the polymer solution. The three types of inorganic fillers selected were: unsupported Cu NPs, Cu-exchanged in the layered silicates, and Cu-exchanged in the 3D zeolites. The Cu-containing fillers were dispersed in 2 mL of water prior to their introduction in the polymer blend solution, stirred for another 24 h, and degassed in an ultrasonic bath before casting on a glass plate. The solutions were left to dry at room temperature in a fume hood. Once the solvent was fully evaporated, a post-treatment was applied to the membranes for their activation, by immersing them in a 1 M NaOH solution for 1 h and afterwards rinsed at least 3–5 times with plenty of ultrapure water till pH was around 5.5.

### 2.4. Membrane Characterization

The viscosity of polymeric solutions was measured by a viscometer Fungilab (Barcelona, Spain) at 25 °C.

The real copper content of the inorganic fillers was measured by atomic absorption in a 3110 Perkin Elmer spectrophotometer (Madrid, Spain).

Membrane thicknesses were measured using a digital micrometer IP-65, with an accuracy of 0.001 mm (Mitutoyo Corp., Kawasaki, Japan) at several points of each membrane sample.

Water uptake (WU) was calculated using the Equation (1) that takes into account the high hydrophilic character of PVA in the hydrophilicity of the hybrid membranes [24].

(1) $$\mathrm{WU}\left(\%\right)=\frac{W_{wet}-W_{dry}}{\frac{W_{wet}-W_{dry}}{1.0}+\frac{W_{dry}}{1.3}}\cdot 100$$

where $W_{wet}$ and $W_{dry}$ were the wet and dry weight of the membrane pieces, respectively, 1.0 and 1.3 were the correction factors for water and PVA densities, respectively. The three samples were weighted after being immersed for 24 h in water to obtain *W_wet_*. The excess of water from the membrane surface was removed by tapping with filter paper. Samples were left to dry at room temperature and then weighted for the measurement of *W_dry_*.

Ion-exchange capacity (IEC) is defined as mmol of ion exchangeable groups per 1 g of membrane [25]. This property was determined by an acid-base back-titration procedure, slightly modified from the previously used method in our laboratory to increase reproducibility and reduce the risk of dissolution and CO_2_ contamination [10,26]; no differences were observed by lengthening the OH^−^ activation step from 1 to 24 h in the results obtained. The OH^−^ activated samples were immersed in 0.01 M HCl (20 mL) for 24 h to exchange their mobile anions with Cl^−^. The HCl solution [25] was back-titrated with a standardized 0.01 M NaOH solution using phenolphthalein as indicator and the IEC was calculated by Equation (2) as follows:

(2) $$\mathrm{IEC}\left(\mathrm{mmol}/g\right)=\frac{\left(V_{NaOH}-V_{NaOH\;i}\right)\cdot C_{NaOH}}{W_{dry}}$$

where $V_{NaOH}$ was the volume of NaOH spent in the titration of 10 mL of HCl solution and $V_{NaOH\;i}$ was the volume of NaOH spent in the titration of 10 mL of the HCl solution after the anion exchange with the membrane. $C_{NaOH}$ was the molar concentration of the solution employed for the titration. WU and IEC were measured for three different membrane samples to assure reproducibility.

Anion conductivity was measured by electrochemical impedance spectroscopy (EIS) [27], using a VMP3 multichannel potentiostat-galvanostat (Biologic, Seyssinet-Pariset, France). A membrane sample of 1.13 cm^2^ was placed between the electrodes. The impedance experiments were measured over the 3 MHz–100 Hz frequency range at open circuit potential. Experiments were carried out at a controlled temperature of 25 ± 3 °C. Before each measurement, the membranes were activated again in 1 M NaOH solution and rinsed thoroughly with ultrapure (18.2 MΩ) water. Anion conductivity was calculated by Equation (3),

(3) $$\sigma =\frac{L}{Ra}$$

where L was the membrane thickness (cm), a the surface of the membrane exposed to the electric field (cm^2^), and R the membrane resistance (Ω) obtained from Nyquist and Bode diagrams (not shown).

Thermogravimetric analyses (DTA-TGA) were performed with a thermobalance (DTG- 60H, Shimadzu, Kyoto, Japan) in air and N_2_ (50 mL/min). Membrane samples between 1 and 5 mg were placed in an alumina pan. The samples were heated up to 650 °C at a rate of 10 °C/min in air and in nitrogen. The water content (WC) bound to the polymeric matrix was determined from the TGA curves, expressed in wt %. Two values of mass were taken from the curves, *m*_1_ and *m*_2_, corresponding to temperatures $T_{1}$ and $T_{2}$ (the range of temperature in which a remarkable drop in mass was observed, between 119 °C and the decomposition temperature). WC was calculated using the equation reported by Franck-Lackaze et al. [28],

(4) $$\mathrm{WC}\left(\%\right)=100\cdot \left(1-\frac{m_{1}}{m_{2}}\right)$$

Water vapor permeability (WVP) was measured following the ASTM E96-05 method, as modified by McHugh et al. [29], and described by Hosseini et al. [30]. This method consisted in sealing the membrane sample between the openings of two glass cups with a diameter of 39 mm, one of them was filled with 6 mL of distilled water, which is equivalent to a 100% RH at 20 °C. The system was placed in a desiccator at room temperature and 0% RH. The water that permeated through the membrane was controlled by measuring the weight of the system every 2 h, until 10 h. Weight loss was plotted versus time, and the slope obtained (*R*^2^ > 0.99) was divided by the membrane exposed area to calculate the water vapor transmission rate WVTR (g/h m^2^). WVP was calculated by applying the following equation

(5) $$\mathrm{WVP}\left(g\;\mathrm{mm}/{\mathrm{kPa}\;h\;m}^{2}\right)=\frac{\mathrm{WVTR}\cdot L}{∆p}\;$$

where ∆*p* was the water vapor pressure difference (kPa) between the two sides of the membrane, which was equal to 2.337 kPa for the given difference in RH between both sides and *L* was the membrane thickness in mm. The measurement of this property was performed twice for each membrane sample.

TEM images of selected membrane samples were observed using a TECNAI T20 microscope (Instituto de Nanociencia de Aragón, Zaragoza, Spain) operating at 200 kV. Membranes were fixed in epoxy resin and cut with an Ultramicrotome (Leica EM UC7, Instituto de Nanociencia de Aragón, Zaragoza, Spain) equipped with a diamond knife. A 50 nm thick slice was deposited on a holey carbon copper grid before electronic observation.

The mechanical resistance of the membranes was measured by the tensile strength and the elongation at break in a Universal Testing Machine (Instron 8874, (Instituto de Nanociencia de Aragón, Zaragoza, Spain) with a head load up to 5 kN and an axial velocity of 5 mm/min. An average of 2–7 samples, cut to specified dimensions, per membrane composition, were measured in humid conditions.

The crystalline structure of the samples was investigated by means of room temperature XRD. The patterns were collected on a Philips X’Pert PRO MPD diffractometer (CITIMAC-Universidad de Cantabria, Santander, Spain) operating at 45 kV and 40 mA, equipped with a germanium Johansson monochromator that provides Cu Kα_1_ radiation (λ = 1.5406 Å), and a PIXcel solid-angle detector, at a step of 0.05°.

XPS were recorded on a K-Alpha Thermo Scientific spectrometer using AlKα (1486.6 eV) radiation, monochromatized by a twin crystal monochromator, and yielded a focused X ray spot with a diameter of 400 mm, at 3 mA and 12 kV. Deconvolution of the XPS spectra was carried out using a Shirley background.

In order to obtain a more detailed speciation of the metal loading in the membranes X-ray absorption spectra were acquired at Cu K-edge (8905.3 eV) at CLAESS, the X-ray absorption spectroscopy beamline of ALBA synchrotron (Barcelona, Spain) [31]. Data normalization and linear combination were done using the Athena free software (Demeter 0.9.25)) [32].

### 2.5. Analysis of Variance: Effects of the Preparation Variables in Membrane Properties

To study the possible effect and interaction of the preparation variables in the properties measured for the membranes, a statistical analysis was performed on the operation conditions considered in this work. The experimental factors analyzed were the type of filler, the filler loading, and the Cu content in the membrane matrix. The properties studied in this analysis were: IEC, conductivity, WU, WVP, thickness and mechanical properties, TS, and elongation at break. The 3-factor analysis of variance (ANOVA) was applied to all the replicate data measured for the different properties to analyze the interaction between them. The effects of the filler type and loading along with the Cu content in the membrane on the properties measured in this work were determined by comparing the F-statistic with its critical value, with a 95% confidence level (*α* = 0.05).

## 3. Results and Discussion

### 3.1. Physico-Chemical Characterization of the Membranes

Table 1 contains the list of the membranes studied in this work. The viscosity of the dispersion of Cu/CS:PVA solution was reduced 97% from the pure polymer blend, as presented in Table 1, complicating the casting of these membranes. Consequently, the membranes filled with unsupported Cu NPs, due to the low solution viscosity, had to be cast on plastic 12 cm × 12 cm Petri dishes instead of on glass plates as for the rest of the membranes. The presence of unsupported Cu NPs affected not only the viscosity of the polymeric solution, but also its color. It was remarkable that the two NP loadings studied modified diversely the color of the resulting membrane, being the 5Cu/CS:PVA membrane dark blue while the 10Cu/CS:PVA one was green.

This may be caused by the different oxidation states of Cu depending on its loading and interaction with the polymers and solvents. A blue color is typical of Cu(II), which can be produced due to the oxidation of metallic copper with water and oxygen as reported by De Godoi et al. [15] according to the following reactions,

(6) $$2{\mathrm{Cu}}^{0}{}_{\left(s\right)}+O_{2\left(g\right)}+2H_{2}O\to 2{\mathrm{Cu}}_{\left(\mathrm{aq}\right)}^{+2}+4{\mathrm{OH}}_{\left(\mathrm{aq}\right)}^{-},$$

(7) $${\mathrm{Cu}}^{0}{}_{\left(s\right)}+2H_{2}O\to {\mathrm{Cu}}_{\left(\mathrm{aq}\right)}^{\;+2}+H_{2\left(g\right)}+2{\mathrm{OH}}_{\left(\mathrm{aq}\right)}^{-}$$

**Figure 1:**
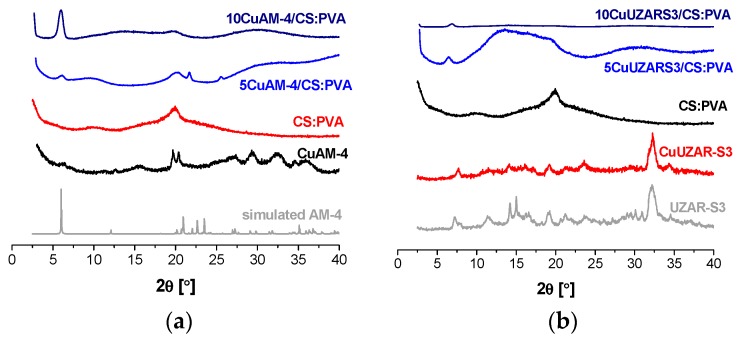
XRD diffractograms of layered CuAM-4 titanosilicate-filled (**a**) and CuUZAR-S3 stannosilicate filled (**b**) CS:PVA mixed matrix membranes.

On the other hand, the color of the membranes with layered silicates and zeolites could also indicate the Cu content and oxidation state. When Cu was ion-exchanged in AM-4 and UZAR-S3, the color of the resulting derived powder and the membranes was blue, while those exchanged in the Y, MOR, and BEA zeolites did not reveal any coloration. This agreed with the real copper content determined by atomic absorption spectrometry, and also shown in the last column of Table 1. In general, the Cu content of these membranes agreed with the filler loading, except for the CuAM-4-based membranes, which showed very high Cu contents due to the higher adsorption capacity as reported elsewhere [20]. This high Cu cation exchange capacity also produced a slight shift of the X-ray diffraction pattern of CuAM-4 compared to the simulated AM-4 obtained from crystallographic data [33], as represented in Figure 1a.

The X-ray diffractograms in Figure 1 showed the interaction between the layered silicates and the CS:PVA polymer blend at 5 and 10 wt % values of filler loading. As previously observed, the broad bands of CS and PVA at 10 and 20° were discerned in the CS:PVA blend membrane and diffuminated upon increasing the layered silicate loading, which could then be attributed to the filler dispersion in the membrane matrix and partial separation of the inorganic layers [32].

**Figure 2:**
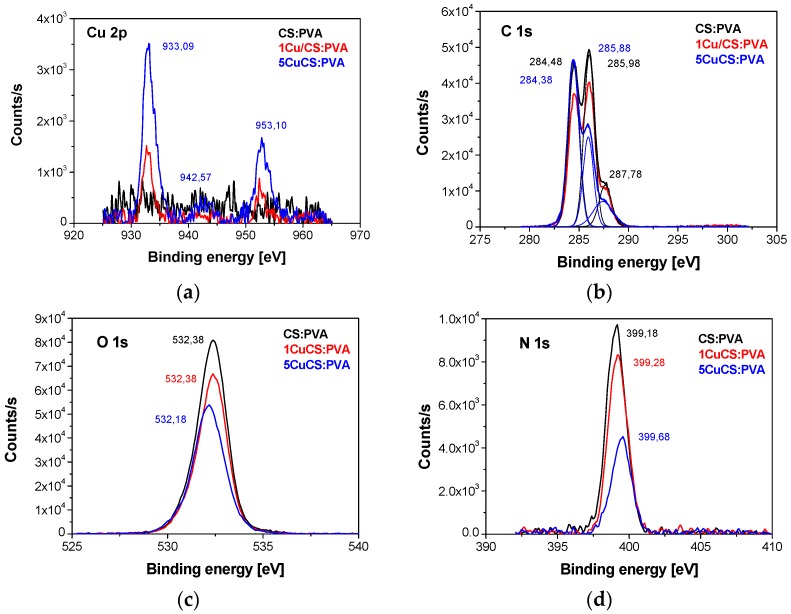
XPS spectra of Cu 2p (**a**); C 1s (**b**); O 1s (**c**) and N 1s (**d**) for the Cu/CS:PVA membranes.

XPS has been often reported to determine the oxidation state of the Cu cations after sorption in CS:PVA membranes considering that CS can reduce the metal cations [34]. Thus, to have first insight on the interaction of copper with the CS:PVA polymeric matrix. XPS analyses were conducted on the membrane prepared with the unsupported Cu NPs and is represented in Figure 2. Cu (II) in CS:PVA blend nanocomposites seemed to be present in the form of 5 different species, Cu^2+^, Cu(OH)^+^, Cu(OH)_2_, Cu(OH)^3+^, and Cu(OH)_4_^2−^ as a function of increasing pH [35]. The summary of the XPS assignments were given in Table S1 in the Supporting Information section.

**Figure 3:**
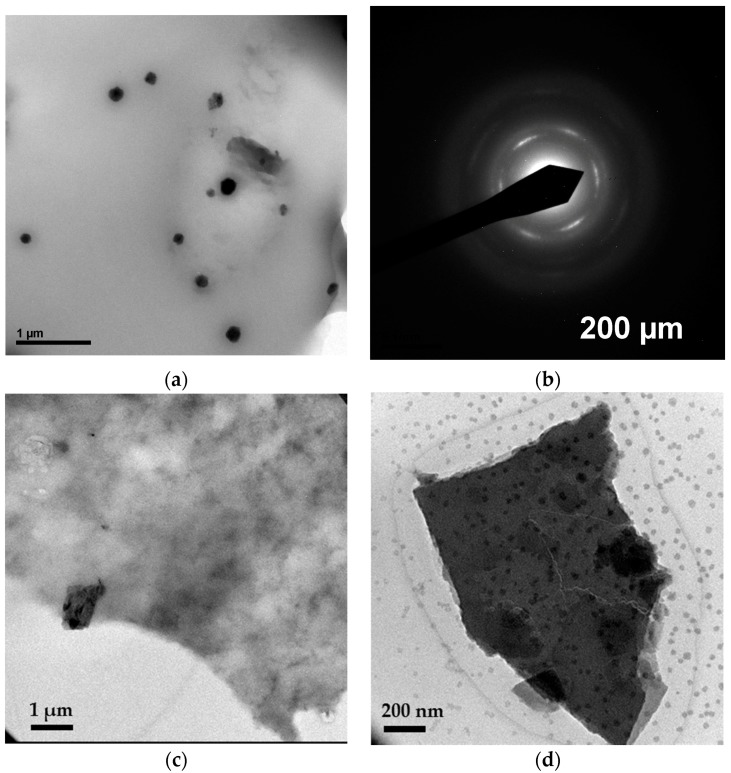
TEM images of MMM 5Cu/CS-PVA (**a**) and the electron diffraction to a single Cu NP (**b**); TEM images of 5CuAM-4/CS:PVA membrane (**c**) and a detail of 5CuUZAR-S3/CS:PVA membrane (**d**).

Further insight into the Cu dispersion into the CS:PVA matrix of selected membrane materials was provided by TEM. The TEM image in Figure 3a revealed a homogeneous dispersion of the CuNPs in the CS:PVA matrix, being the size of the NPs 33.5 ± 0.7–15.8 ± 1.4 nm. The rings in the electron diffraction pattern of the polycrystalline metal NPs in Figure 3b could then be indexed as (0 2 0) and (1 3 1) of CuO and Cu(OH)_2_, respectively. This agreed with the literature on Cu oxidation states, where the Cu was transformed to Cu(OH)_2_ upon ion exchange in 1M NaOH, providing the blue color and further transformations to anion intermediate blue-green species. The change from blue to green color was caused by the reduction of Cu(II) to Cu(I), and the formation of Cu_2_O (green) and CuO (black-brown) species [35]. These patterns were similar to those observed by Domenech et al. for Nafion 117 membranes modified with Ag NPs [36], in the stabilization of CuO by PVP [37] and in the CS-capped copper oxide nanopaint preparation [14].

The TEM image of the CS:PVA membranes with a 5 wt % Cu exchanged in layered titanosilicate AM-4 presented in Figure 3c revealed the abundant dispersion of the filler in the polymer blend. A detail of a single layered UZAR-S3 particle in a 5 wt % CuUZAR-S3/CS:PVA membrane was shown at higher magnification in Figure 3d, where the lamella was surrounded by Cu NPs in a similar way as in the titanosilicate filler [20]. This proved the interest of supporting the copper in a porous inorganic filler that could ease the fabrication of a homogeneously dispersed metal within the polymeric matrix.

**Figure 4:**
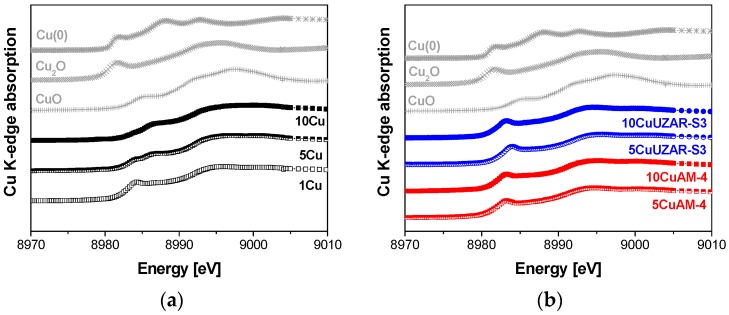
Normalized Cu K-edge XANES spectra for CS:PVA membrane samples filled with unsupported Cu NPs (**a**) and Cu-exchanged layered titanosilicate AM-4 and stannosilicate UZAR-S3 (**b**). References used for the linear combination (Cu foil, Cu_2_O, and CuO) are also shown above.

In order to obtain a more specific analysis and ensure the bulk chemical composition of Cu-containing CS:PVA membranes, which is not the case for XPS, X-ray absorption synchrotron measurements [31] were carried out, and the normalized energy spectra of the samples were compared with the standard spectra of pure standards, namely, metallic copper foil (Cu(0)), and inorganic oxides Cu_2_O (Cu(I))and CuO (Cu(II)) oxides, as represented in Figure 4. The edge positions, defined as the maximum point of the first derivative function in the rapidly rising edge step of the absorbance vs. energy plot, have been found to be 8979.0, 8980.6, and 8983.6 eV for Cu foil, Cu_2_O, and CuO, respectively [38]. By direct observation of Figure 4a, we could estimate that Cu in unsupported CuNPs/CS:PVA membranes looked evolving from a majority of Cu(II) (10Cu) towards Cu(I) since the rising edge is shifting towards higher energies with increasing filler loading. At higher Cu content (10Cu and 5Cu), a double peak structure of the first derivative of the XANES spectra suggested coexistence of Cu^1+^ and Cu^2+^ species. In addition, the global XANES shape evolution in the 8982–8990 eV energy range was directly suggesting the formation of [CuOH]^+^ and Cu(OH)_4_^2−^, as a function of increasing pH [35], as estimated from TEM and XPS analyses above. In the case of the Cu-exchanged layered silicates/CS:PVA membranes (Figure 4b), the XANES spectra were typical of Cu(I) sites, accounting for their exchange in the porous inorganic structure [39,40] and the favored interaction of copper ions with the amorphous part of CS in the CS:PVA matrix, forming a coordinated bridge between NH_2_ and OH [16]. Despite the ongoing challenge for EXAFS and XANES analysis, from XANES spectra in Figure 4 a structural compression could be hypothesized with respect to the reference samples. This agreed with the literature on copper-exchanged zeolites where the feature around 8983 eV, corresponding to Cu(I) species, is not affected, being that the Cu was not oxidized in the presence of water [40]. Therefore, the results of the XANES/EXAFS spectra agreed with previous observations regarding the stability of the Cu in the supported inorganic fillers in comparison with the unsupported Cu NPs in the CS:PVA blend polymer matrix.

The WU, IEC, and OH^−^ conductivity of the prepared membranes in OH^−^ form were shown in Table 2. As expected, the addition of Cu NPs decreased the WU of the CS:PVA membranes, as reported for Ag NPs in Nafion 117 [6]; in CS [14] IEC increased, with 5 wt % as the apparent optimum Cu NPs loading. This was attributed to the different oxidation states of Cu we shown above. As expected, the addition of Cu NPs decreased the hydrophilicity of the membranes. On the other hand the optimum filler loading for the layered silicate seemed to be 10 wt %, since the IEC of the membranes with 10 wt % CuAM-4 and CuUZAR-S3 was higher than those reached at 5 wt % loadings. In fact, the values obtained (0.464 mmol/g for the 10CuAM4/CS-PVA membrane and 0.451 mmol/g for the 10CuUZAR-S3/CS-PVA membrane) were above the value measured for the commercial FAA-3 membrane (0.364 mmol/g).

**Figure 5:**
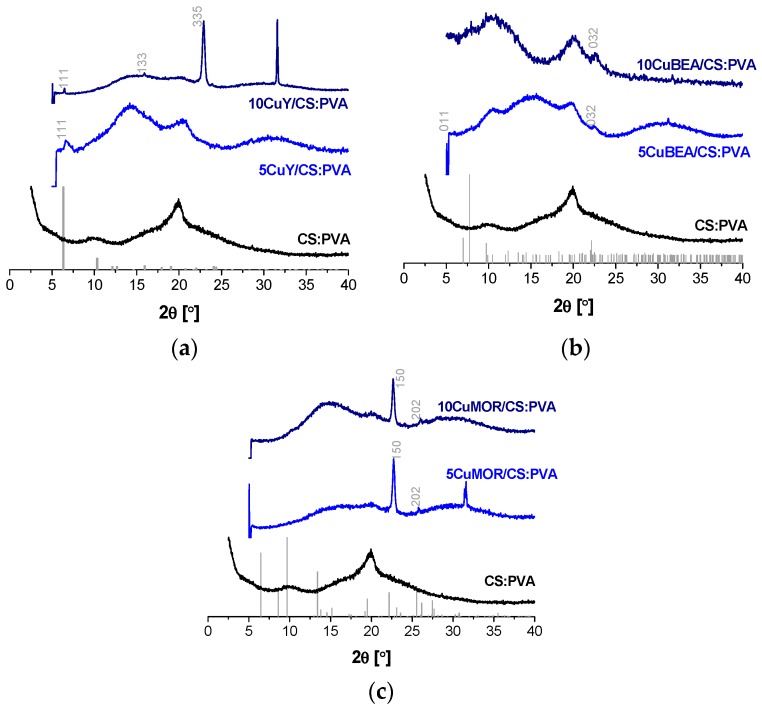
XRD diffractograms of CuY (**a**); CuBEA (**b**); and CuMOR (**c**) filled CS:PVA MMMs. The simulated patterns were taken from the crystallographic data of the International Zeolite Database (www.iza-structure.org/databases/).

WU was reduced by the introduction of the Cu-based fillers, except for the layered silicates whose hydrophilic character was larger than the ones provided by the other type of fillers. This confirmed the hydrophilic character of this kind of filler [41]. In fact, a large amount of water-induced swelling was observed for the Cu exchanged in layered silicates-filled CS:PVA membranes, in detrition of the mechanical properties of these membranes. The particular case of the membranes with Cu exchanged on AM-4 showed that this kind of filler tended to increase the WU values. The high hydrophilic character of the titanosilicate surpassed the capacity of Cu to control this property and thus, by increasing the amount of filler, the WU also increased. Then, Cu-exchanged zeolites Y, MOR, and BEA-filled membranes were also prepared. Although Cu-exchanged zeolites were very difficult to observe under TEM, because of the resistance opposed by the 3D particles to the diamond knife upon sample preparation, and due to the lower homogeneity in their dispersion through the membrane matrix, the XRD diagrams in the zeolite filled membranes corroborated the presence of CuY, CuBEA, and CuMOR in Figure 5a–c, respectively. As expected, the relative intensity of the CS and PVA main peaks diminished upon increasing loading of the crystalline zeolite content [41], despite the presence of some impurities in the sample holder in some samples (33° peak in Figure 5a,c). They presented lower WU values than the pristine polymer membrane and the ones retrieved for the membranes filled with CuAM-4 and CuUZAR-S3, especially those filled by CuMOR, regardless the filler loading. The IEC of Cu-exchanged zeolite filled CS:PVA MMMs also showed similar values at 5 and 10 wt % filler loadings, even surpassing the IEC value of the FAA-3 membrane in the case of 5CuMOR/CS:PVA membrane. These observations led to the conclusion that it may be the type of filler and not the Cu content that was influencing the physicochemical properties of the membranes.

Likewise, the OH^−^ conductivity followed a trend similar to that of the IEC. As conductivity depended on both IEC and WU, it was therefore influenced by the type of filler. Maximum conductivity values were reached at 10 wt % loading for the Cu ion-exchanged layered AM-4 and UZAR-S3, although CuMOR also showed a relevant improvement. The highest OH^−^ conductivity was obtained for the membranes where Cu was ion-exchanged in the layered UZAR-S3 and AM-4 and the zeolites Y and MOR, as shown in Table 2, although still far from the commercial FAA-3 membrane measured in the same way. This comparison with the commercial membranes in the same measurement conditions, as well as checking the whole membrane region by measuring at different pieces of each membrane sample, have proved the reliability of our measurement in alkaline conditions, although it is true that anion-exchange membranes in OH^−^ form exposed to ambient air in humid conditions may undergo the partial transformation of OH^−^ to bicarbonate and carbonate anions that would mask the measurement of OH^−^ conductivity [26]. The stability of CS:PVA based membranes in alkaline media has been corroborated previously when similar efficiencies to the commercial Fumatech membrane were obtained, though [42]. Details on the Cu state in the membrane framework could be observed in the FT-EXAFS spectra in Figure S2 (Supporting Information).

The relationship between IEC and WU for all the membranes prepared with the layered fillers, CuAM-4 and CuUZAR-S3, showed a linear dependence with adjustable *R*^2^ larger than 89% and a significant adjustment (*p* < 0.05). That was the reason for producing the membranes with 15 wt % filler loading in these cases, at this point. However, a filler loading of 15 wt % did not improve the IEC or the OH^−^ conductivity any further. Thus, the general optimum value of inorganic filler loading seemed to be 10 wt %. A higher amount of inorganic particles in the polymeric matrix probably hindered the mobility of anions and water molecules through the membrane, without altering the oxidation state of Cu in the membrane [43].

In addition, by looking at Figure 4b, we observed that when the Cu oxidation state looked lower (main feature at lower energy), the conductivity and IEC in Table 2 were higher. This revealed a correlation between the Cu oxidation state and the electrochemical functional properties.

The correct correlation between water content and water vapor permeability (WVP) may establish new AEM designs for electrochemical applications [3], taking into account that an excessive WU can also lead to dilution of the groups allowing the water and ions transport and, consequently, a reduction of the anion conductivity [44]. As a consequence, there is a trade-off between these membrane properties, which is the reason why it was interesting that the anion conductivity of the 10CuAM-4/CS:PVA membrane increased without a great change in the WU, although the conductivity of the FAA-3 commercial membrane was not surpassed. As shown in Table 2, the WVP varied with the inclusion of the Cu-exchanged particles and the water sorption capacity of the inorganic filler. Every type of filler support for Cu used in this work, except for the unsupported CuNPs and CuBEA, acted as a water barrier through the polymeric matrix, reducing the permeability compared to the membrane without the inorganic filler added. The values obtained were in the same of order of magnitude as other equimolar CS-alginate blends reported in the literature [45]. Those fillers of superior hydrophilic character, like CuY and CuMOR, showed a significant (*p* < 0.05) relationship between WVP and WU, with a negative relationship, which meant that the WVP decreased with decreasing WU in the membrane. This was due to the water retention in the interstitial spaces between the polymer chains and the inorganic fillers [46]. This phenomenon did not occur for CuNPs or CuBEA—filled CS:PVA membranes, which could explain why WVP increased when adding these highly hydrophobic fillers.

**Figure 6:**
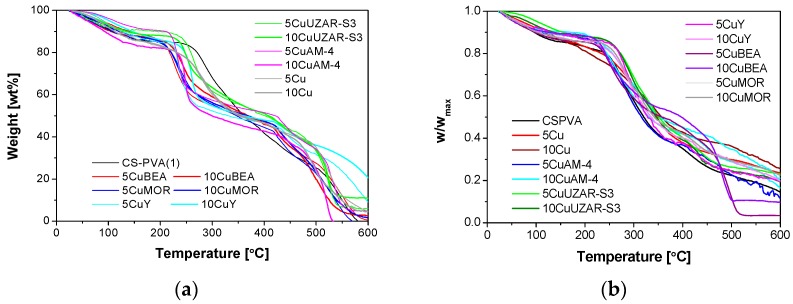
Thermal decomposition of the CS:PVA-based membranes under air (**a**) and nitrogen (**b**).

The thermogravimetric curves of the CS-PVA-based MMMs in air and nitrogen are displayed in Figure 6a,b, respectively. The shape of the curves was very similar to previously reported TGAs for CS:PVA blends [10], no matter what type of filler was used. The same characteristic weight losses for CS and PVA in the CS:PVA blend membranes were observed, as reported in a previous work [10]. The first weight loss at 119 °C represented the free water content, which is the difference between the excess of water after rinsing and immersing the membrane for the measurement of the WU and the bound water determined by the hydrophilic character of the polymer [47]. The second weight loss corresponded to the polymer chain degradation and varies between 246 and 334 °C for all the membranes. The weight loss at 119 °C and at the thermal decomposition, T_2_, calculated from the TGA curves in N_2_ in Figure 6b were given in Table S2. These values were close to the thermal decomposition temperature of CS membranes (329.5 °C) having a weight loss of 8.95 wt % [45]. The decomposition temperature varied, but it is near 200 °C for every membrane. The different peaks through this phase of decomposition could be attributed to the degradation influence of the different types of fillers on the degradation of the polymer matrix. The average residual weight values of the membranes were 5 ± 0.35, 5.31 ± 0.47, 10.54 ± 0.05, 4.7 ± 0.06, 6.04 ± 0.4, and 7.21 ± 0.28 wt % for the Cu/CS:PVA, CuUZAR-S3/CS:PVA, CuAM-4/CS:PVA, and CuY/CS:PVA membranes, respectively, which, comparing with the nominal weight of the inorganic fillers (5, 10, 15 wt %), could signify a rather heterogeneous distribution of the particles throughout the membrane matrix. This heterogeneous distribution was clearly observed with the naked eye for CuBEA and CuMOR filled CS:PVA membranes, which probably explained the differences in the TGA curves in Figure 6 compared with the values displayed by the other membranes.

For stability analysis, the WC, the bound water content of the AEMs was calculated by Equation (6), following the method of Franck-Lackaze et al. [28] (Table S2). Although the WC of the pristine CS:PVA membrane agreed with that obtained previously [10], the WC of layered CuAM-4/CS:PVA and CuUZAR-S3/CS:PVA membranes was higher than the values obtained previously for the unexchanged AM-4 and UZAR-S3/CS:PVA membranes [10]. This could be attributed to the success of the Cu-exchange in the silicate layer structure prior to the preparation of the membranes. The WC increased upon addition of CuAM-4, CuUZAR-S3, and CuY, and decreased when adding Cu NPs up to 5 wt %, CuMOR and CuBEA, as an indicator of the higher hydrophobic character of the latter type of fillers, compared to the formerly mentioned ones. As a conclusion, the thermal decomposition temperature decreased with the hydrophilic filler loading, and increased slightly with the hydrophobic filler content.

### 3.2. Mechanical Properties

The Cu-exchange has been reported to decrease the tensile strength (TS) of Neosepta membranes from 2.67 ± 0.75 kg/m^2^ to 2.26 ± 0.39 kg/m^2^ [48]. Metal nanoparticles have not been observed to improve the TS performance of hydrophilic Nafion membranes either [6]. The results of 2–7 repetitions of the TS experiments performed for every CS:PVA membrane sample in this work are collected in Table 3. The TS of uncross-linked CS has been reported as 1.38 N/mm^2^, in the order of magnitude of the TS values observed in this work (Table 3) although the values for organically cross-linked CS:PVA membranes under dry conditions given in the literature were around 65.53 ± 6.9 N/mm^2^, with an elongation at break of 11.05% ± 1.3% [11,12,49]. The overall trend of TS of the membranes in this work was lower than those previously obtained, those were obtained on cross-linked membranes using glutaraldehyde and trimesoyl chloride, which also reduced WU and anion exchange capacity [50], in contrast with our membranes.

Nevertheless, the metal supported in layered AM-4 and UZAR-S3, as well as in zeolites Y and MOR, showed a slight improvement in the TS under wet conditions, compared to the pure CS:PVA blend polymer membrane. However, the membranes filled by unsupported Cu, as well as high loading UZAR-S3, experienced high degree of swelling.

The mechanical properties could thus be related to the ion exchange capacities shown in Table 2, since the higher the IEC, the lower the TS values, as reported for other AEMs [24].

### 3.3. ANOVA-Based Statistical Analysis

A statistical analysis of all the measured properties (WU, IEC, conductivity, WVP, thickness, TS, and elongation at break) was carried out to confirm the observations presented in the previous sections. The ANOVA analysis was performed to determine which factors (type of filler, filler loading, or Cu content) and how, have a statistically significant effect on each parameter. These variables were selected because already Kikhavani et al. [51] observed that the ratio filler/polymer had an effect on the accessibility to the surface of inorganic particles used as fillers that affected the IEC and conductivity of AAEMs. The polymer blend ratio or the solvent used had been observed to have an effect in water sorption (WU) [11]. The effect of the type of filler was evaluated qualitatively.

The results of the ANOVA analyses for the different parameters with significance (*p* < 0.05) in all or some of the factor contributions are shown in Table 4, Table 5, Table 6 and Table 7, while the rest can be found in Tables S3–S5 of the Supporting Information. The ANOVA in Table 4 indicated that while the thickness of the membranes depended significantly on all the three input factors (type of filler, filler loading, and copper content), according to results in Table 5, WU varied as a function of the type of filler and Cu content, with a lesser dependence in filler loading, which agreed with the observations discussed earlier in this work, while the elongation at the break in Table 7 only depended on the type of filler and filler loading. Contrarily, the tensile strength in Table 6 depended mainly on the filler loading, regardless the copper content and the type of filler support.

**Figure 7:**
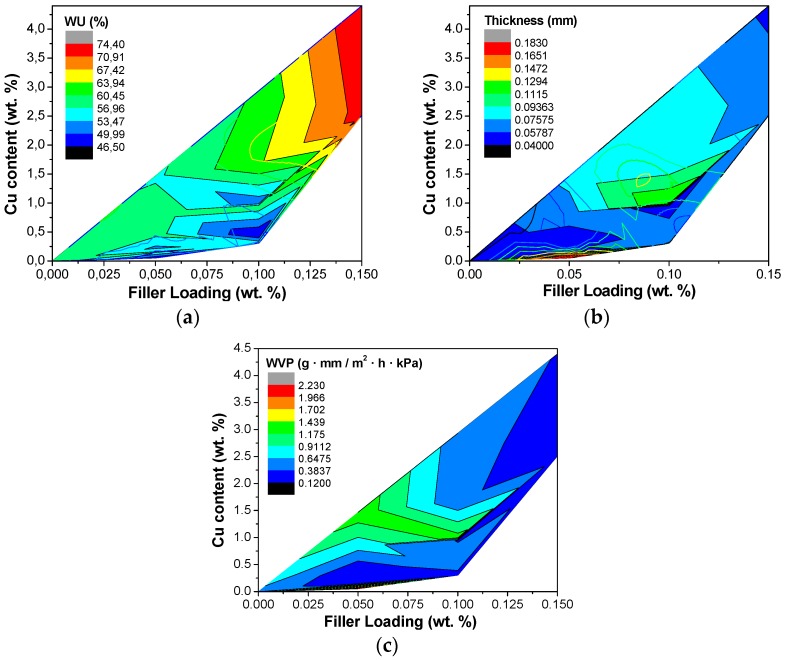
Contour plots of the regression models obtained for parameters WU (**a**); thickness (**b**) and WVP (**c**).

In this work, a first order linear model with interactions was considered for the parameters WU and thickness. In each case, only the factors and interactions that have a statistically significant effect on the property, as determined above, were included in the regression model. The surface responses thus obtained for each model parameter were plotted qualitatively in the contour plots in Figure 7, for the hydrophilic properties WU and WVP, and the thickness, because of its influence in WVP, as a function of filler loading and copper content.

The hypotheses of independence, statistical significance, normality, and homoscedasticity have to be met in order to confirm the validity of the ANOVA analysis [52]. On the one hand, the homoscedasticity was checked by the studentized Breusch–Pagan test, with a confidence level of 95%. On the other hand, the normality hypothesis was verified using the unidimensional statistics of the ANOVA residuals, by the Shapiro–Wilk and Jarque–Bera tests. Nevertheless, these validations were not shown here for the sake of simplicity [53].

Figure 7 confirmed that the WU increased with increasing Cu content and filler loading in the CS:PVA based membranes (Figure 7a), while the thickness and WVP only increased up to a maximum as observed in the previous section, in agreement with the literature [53]. In addition, it was observed that the influence of the factors filler loading and Cu content with a statistically significant secondary effect on the other properties (IEC, conductivity, TS, and elongation) like WVP with the thickness, could be estimated from, by considering the contribution of the primary factors on the thickness and WU. These qualitative model fitting data are reported in the Supporting information section.

## 4. Conclusions

In this study, the preparation of AAEMs from renewable and low-cost polymers has been optimized, as a function of the type of inorganic filler support, filler loading, and Cu content, regarding different properties (IEC, WU, WVP, thickness, TS, elongation at break, and conductivity). The copper content was introduced in the polymer CS:PVA blend matrix either as unsupported commercial nanoparticles, or ion-exchanged in various porous supports like AM-4, layered UZAR-S3 stannosilicate, and zeolites Y, MOR, and BEA. The WU, IEC, and anion conductivity can be controlled by the addition of the metal particles, by altering the hydrophilicity and ion and water sorption and permeability of the hydrophilic polymer blend. In contrast, chemical, thermal, and mechanical resistance are better controlled when the metal is exchanged in a porous inorganic matrix. Moreover, fillers of hydrophilic nature, i.e., CuAM-4, CuUZAR-S3, and CuMOR, showed the best results in ion exchange and conductivity.

In order to find the optimum conditions as a compromise between the electrochemical and water management properties, a three variable factors ANOVA was conducted to identify the optimum values of the three factors. The optimum filler loading seemed to be 10 wt %. The predicted values for WU, WVP, and thickness were confirmed experimentally with high precision. The results from this analysis give scope for a further optimization and development of novel anion-exchange hydrophilic membranes for electrochemical applications, or even as antifouling membrane coatings in water treatment, although a significant amount of work is still to be carried out regarding the evaluation of carbonation and influence of different anion species in the polyelectrolyte membrane performance.

## Figures and Tables

**Table 1:** Description of the membranes prepared.

Membrane ^1^	Filler Loading (wt %)	Viscosity (mPa s)	Color of Solution	Color of Membrane	Cu (wt %)
CS:PVA	0	223	Yellowish	Transparent	0
1Cu/CS:PVA	1	19.8	Grey/green	Light blue	1
5Cu/CS:PVA	5	7.80	Grey/green	Dark blue	5
10Cu/CS:PVA	10	4.50	Grey/green	Green	10
5CuUZAR-S3/CS:PVA	5	120	Light blue	Blue	1.47
10CuUZAR-S3/CS-PVA	10	114	Light blue	Blue	2.93
15CuUZAR-S3/CS:PVA	15	112	Light blue	Blue	4.40
5CuAM-4/CS:PVA	5	126	Light blue	Blue	0.84
10CuAM-4/CS:PVA	10	123	Light blue	Blue	1.68
15CuAM-4/CS:PVA	15	121	Light blue	Blue	2.51
5CuY/CS:PVA	5	117	White	Transparent	0.15
10CuY/CS:PVA	10	110	White	Transparent	0.30
5CuMOR/CS:PVA	5	116	White	Transparent	0.22
10CuMOR/CS:PVA	10	111	White	Transparent	0.45
5CuBEA/CS:PVA	5	120	White	Transparent	0.47
10CuBEA/CS:PVA	10	114	White	Transparent	0.94

^1^ Cu denote the Cu NP fillers, CuAM-4, and CuUZAR-S3, the Cu-exchanged in layered titanosilicate AM-4 and layered stannosilicate UZAR-S3, respectively, whereas CuY, CuMOR, and CuBEA, denote the Cu-exchanged in zeolites Y, MOR, and BEA, as described in the Materials section.

**Table 2:** WU, IEC, and OH^−^ conductivity, and WVP and thickness of the membranes studied in this work.

Membrane	WU (wt %)	IEC (mmol/g)	Conductivity (mS/cm)	WVP (g·mm/m^2^·h·kPa)	Thickness (µm)
FAA-3	23.2 ± 2.9	0.36 ± 0.021	2.92 [28]	1.159	41 ± 1 ^2^
CS:PVA	60.1 ± 2.9	0.27 ± 0.03	0.26 ± 0.06	0.577 ± 0.043	38 ± 4
5Cu/CS:PVA	47.2 ± 2.8	0.37 ± 0.12	- ^1^	2.187 ± 0.073	183 ± 27
10Cu/CS:PVA	51.4 ± 4.3	0.28 ± 0.04	0.218	1.354 ± 0.117	130 ± 32
5CuUZAR-S3/CS:PVA	56.5 ± 3.3	0.27 ± 0.01	0.28 ± 0.00	1.632 ± 0.590	74 ± 7
**10CuUZAR-S3/CS:PVA**	**59.5 ± 1.3**	**0.45 ± 0.05**	**0.71 ± 0.10**	0.427 ± 0.100	83 ± 12
15CuUZAR-S3/CS:PVA	75.4 ± 2.2	0.448 ± 0.01	0.20 ± 0.05	0.375 ± 0.062	57 ± 5
5CuAM-4/CS:PVA	60.8 ± 1.7	0.22 ± 0.02	0.25 ± 0.14	0.466 ± 0.049	71 ± 9
**10CuAM4/CS:PVA**	**64.7 ± 1.2**	**0.46 ± 0.07**	**0.90 ± 0.36**	0.348 ± 0.042	91 ± 11
**15CuAM-4/CS:PVA**	**71.2 ± 2.3**	**0.44 ± 0.01**	**0.46 ± 0.13**	0.330 ± 0.104	62 ± 7
5CuY/CS:PVA	56.8 ± 0.7	0.30 ± 0.03	0.32 ± 0.02	0.333 ± 0.042	77 ± 3
10CuY/CS:PVA	56.3 ± 0.9	0.29 ± 0.02	0.34 ± 0.08	0.290 ± 0.139	52 ± 8
5CuMOR/CS:PVA	49.8 ± 5.6	0.37 ± 0.02	0.18 ± 0.01	0.390 ± 0.147	64 ± 9
10CuMOR/CS:PVA	48.0 ± 4.4	0.34 ± 0.05	0.51 ± 0.16	0.336 ± 0.047	64 ± 6
5CuBEA/CS:PVA	59.8 ± 0.8	0.24 ± 0.02	- ^1^	0.219 ± 0.012	46 ± 10
10CuBEA/CS:PVA	58.3 ± 5.6	0.21 ± 0.02	0.33 ± 0.07	0.287 ± 0.042	53 ± 2

^1^ Conductivity could not be measured due to difficulties upon membrane manipulation. ^2^ Unsupported commercial membrane. Numbers in bold indicate the best results as commented in the text.

**Table 3:** Tensile strength and elongation at break of the wet Cu-filled CS:PVA membranes.

Membrane	TS (N/mm^2^)	e (%)
5Cu/CS:PVA	0.14	1.71
10Cu/CS:PVA	0.11	1.49
5CuUZAR-S3/CS:PVA	4.34 ± 2.07	56 ± 13
10CuUZAR-S3/CS:PVA	1.18 ± 0.23	57 ± 40
15CuUZAR-S3/CS:PVA	0.94 ± 0.09	55 ± 9.5
5CuAM-4/CS:PVA	2.14 ± 0.10	66 ± 0.9
10CuAM-4/CS:PVA	0.34 ± 0.05	79 ± 7.0
15CuAM-4/CS:PVA	0.24 ± 0.11	18 ± 15
5CuY/CS:PVA	3.51 ± 2.48	44 ± 3.1
10CuY/CS:PVA	7.57 ± 4.10	36 ± 8.9
5CuMOR/CS:PVA	0.36 ± 0.03	30 ± 12
10CuMOR/CS:PVA	4.83 ± 1.62	37 ± 11
5Cu-BEA/CS:PVA	0.72 ± 0.25	56 ± 5.9
10Cu-BEA/CS:PVA	0.64 ± 0.24	67 ± 20

**Table 4:** ANOVA for parameter thickness (mm).

Source	Sum of Squares (SS)	Degree of Freedom (df)	Mean Square (MS)	*F*-Value	*p*-Value
*Main effects*					
A-Type of filler	9.73 × 10^−3^	5	1.94 × 10^−3^	57.24	0.017
B-Filler loading	2.84 × 10^−3^	1	2.84 × 10^−3^	83.46	0.012
C-Cu content	8.52 × 10^−4^	1	8.52 × 10^−4^	25.07	0.038
*Interaction*					
A × B	8.27 × 10^−3^	5	1.65·×·10^−3^	48.62	0.020
B × C	1.89 × 10^−3^	1	1.89 × 10^−3^	55.65	0.018
A × B × C	8.26 × 10^−4^	4	2.06 × 10^−4^	6.07	0.146
Residual	6.8 × 10^−5^	2	3.4 × 10^−5^		
Total	0.024	19			

**Table 5:** ANOVA for parameter WU (wt %).

Source	Sum of Squares (SS)	Degree of Freedom (df)	Mean Square (MS)	*F*-Value	*p*-Value
*Main effects*					
A-Type of filler	405	5	81	80.16	*0.012*
B-Filler loading	14.8	1	14.77	10.62	0.062
C-Cu content	246	1	245.9	243.4	*0.004*
*Interaction*					
A × B	96.4	5	19.28	19.08	*0.050*
B × C	100.4	1	100.4	99.39	*0.010*
A × B × C	16.9	4	4.23	4.186	0.202
Residual	2.0	2	1.01		
Total	881.5	19			

Values with *p*-value < 0.05 are highlighted in italic.

**Table 6:** ANOVA for parameter TS (N/mm^2^).

Source	Sum of Squares (SS)	Degree of Freedom (df)	Mean Square (MS)	*F*-Value	*p*-Value
*Main effects*					
A-Type of filler	184	5	37	0.231	0.919
B-Filler loading	110,811	1	11,081	69.55	*0.014*
C-Cu content	571	1	571	3.581	0.199
*Interaction*					
A × B	733	5	147	0.921	0.594
B × C	2221	1	2221	13.94	*0.065*
A × B × C	1910	4	477	2.997	0.266
Residual	319	2	159		
Total	116,749	19			

Values with *p*-value < 0.05 are highlighted in italic.

**Table 7:** ANOVA for parameter elongation at break (%).

Source	Sum of Squares (SS)	Degree of Freedom (df)	Mean Square (MS)	*F*-Value	*p*-Value
*Main effects*					
A-Type of filler	3734	5	746.7	15.21	*0.063*
B-Filler loading	1737	1	1737	35.38	*0.027*
C-Cu content	1	1	0.8	0.016	*0.910*
*Interaction*					
A × B	1436	5	287.1	5.847	0.152
B × C	2318	1	2317	47.20	*0.020*
A × B × C	1419	4	354.8	7.224	0.125
Residual	98	2	49.1		
Total	10,743	19			

Values with *p*-value < 0.05 are highlighted in italic.

## Supplementary Materials

The following are available online at http://www.mdpi.com/2073-4360/10/8/913/s1.
