# Supplementary material for: Preparation and Identification of Optimal Synthesis Conditions for a Novel Alkaline Anion-Exchange Membrane
Source: Polymers (Basel). 2018 Aug 13;10(8):913. doi: 10.3390/polym10080913 (PMC6403638; doi:10.3390/polym10080913)
Supplement: Supplementary file 1 [file polymers-10-00913-s001.pdf]

Preparation and identification of optimal synthesis conditions for a novel alkaline anion-exchange membrane

Aitor Marcos-Madrazo[1], Clara Casado-Coterillo[1,*], Leticia García-Cruz[2], Jesús Iniesta[2], Laura Simonelli[3], María del Mar Encabo-Berzosa[4,5], Víctor Sebastián[4,5], Manuel Arruebo[4,5], Ángel Irabien[1]

[1] *Department of Chemical and Biomolecular Engineering, Universidad de Cantabria, Santander 39005, Spain.*

[2] *Department of Physical Chemistry and Institute of Electrochemistry, University of Alicante, Alicante 03080, Spain*

[3] *CELLS – ALBA Synchrotron Radiation Facility, Carrer de la Llum 2-26, 082090, Cerdanyola del Vallès, Barcelona, Spain.*

[4] *Department of Chemical and Environmental Engineering, Instituto de Nanociencia de Aragón, Universidad de Zaragoza, Zaragoza 50018, Spain.*

[5] *Networking Research Center on Bioengineering, Biomaterials and Nanomedicine, CIBER-BBN, 28029 Madrid, Spain.*

(*) corresponding author: casadoc@unican.es

SUPPORTING INFORMATION

Evolution of Cu exchange in the layered silicates by monitoring pH.

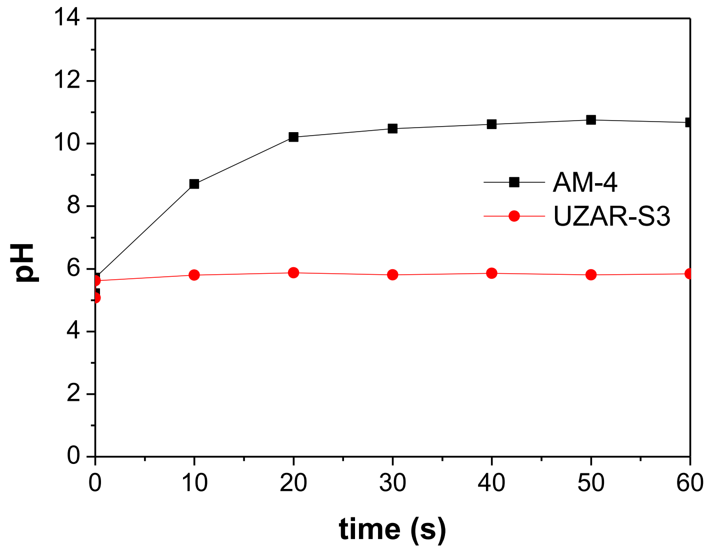

Figure S1. Evolution of pH of the Cu^{2+}solution during the cation exchange process.

Cu speciation observed in the Cu/CS:PVA membranes at different loading by XPS.

Table S1. Deconvolution of XPS spectra for the synthesized membranes and the assignments based on binding energies. At. wt. % are presented between brackets.

**Element**	**CS:PVA(1:1)**	**1Cu/CS:PVA**	**5Cu/CS:PVA**	**Assignment**
C 1s	284.51 (30.73)	284.53 (30.19)	284.54 (41.29)	C-C and Csp^3-H
	286.04 (26.55)	286.07 (26.8)	286.10 (20.21)	C-O or C-N or C-O-C
	287.57 (9.34)	287.61 (9.39)	287.62 (7.20)	C=O or O-C-C
O 1s	531.28 (5.08)	531.00 (3.61)	530.45 (1.61)	C=O

	532.43 (23.32)	532.42 (24.95)	532.29 (25.00)	>C-O
N 1s	399.09 (4.37)	399.25 (4.52)	399.56 (2.61)	$-NH_2$ or NH-
Cu $2p^{3/2}$		932.68 (0.08)	932.8 (0.23)	Cu^{2+}
Cu 2p		933.95 (0.03)	934.54 (0.11)	$Cu(OH)^+$
Cu $2p^{5/2}$		941.44 (0.01)	941.6 (0.02)	$Cu(OH)_2$
Cu 2p		944.28 (0.01)	943.74 (0.02)	$Cu(OH)_3^-$ or $Cu(OH)_4{}^{2-}$

EXAFS synchrotron radiation as a function of the type of filler support.

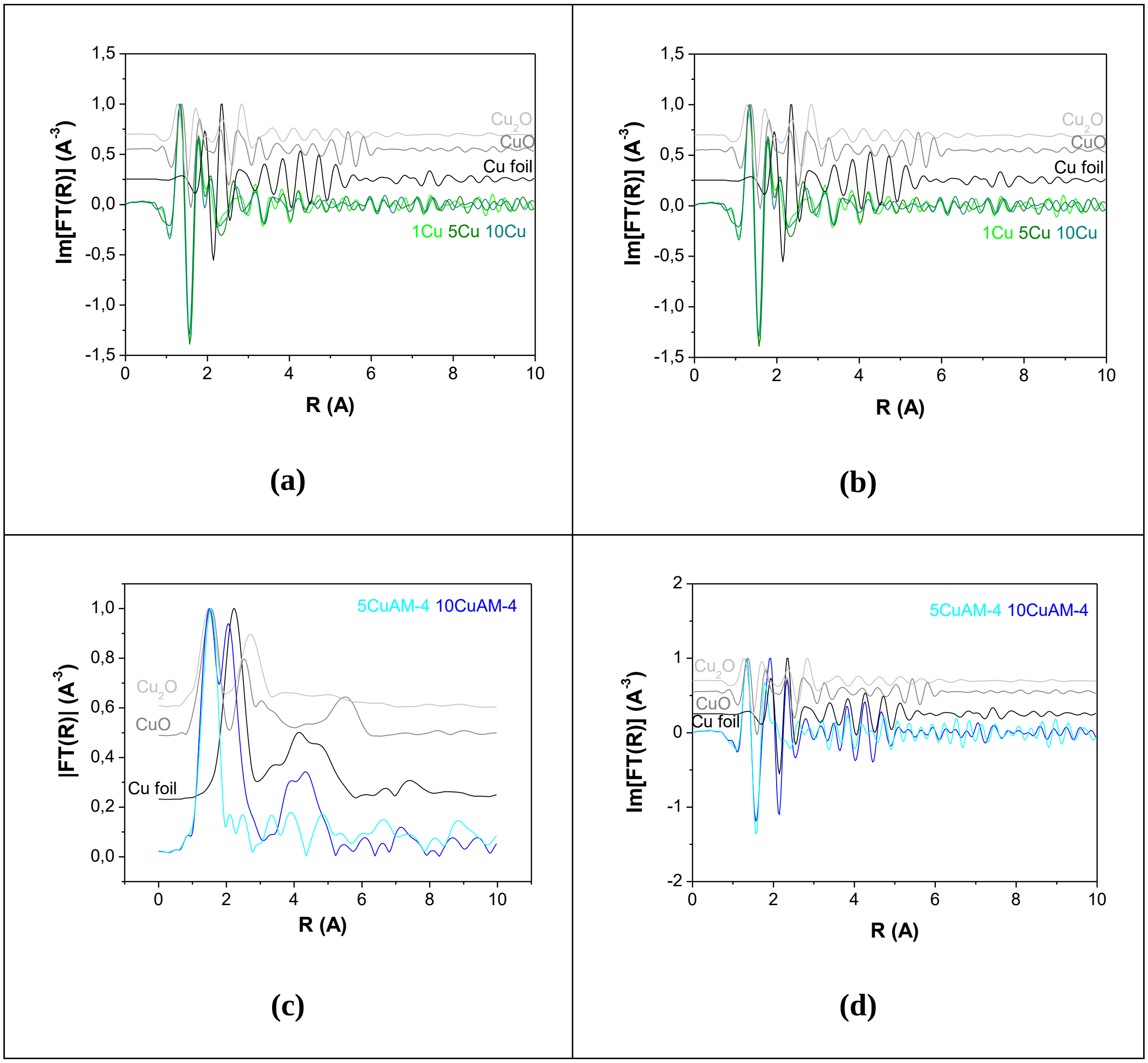

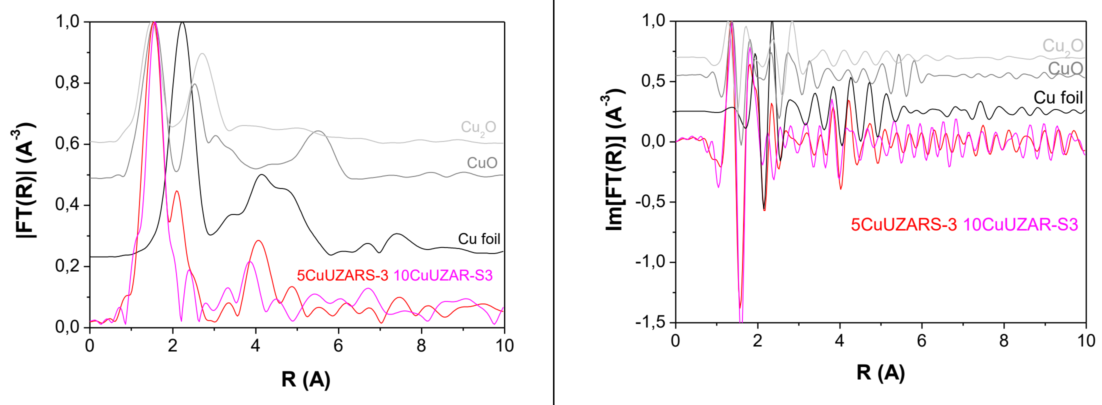

(e) (f)

Figure S2. Magnitude (right column) and imaginary part (left column) of Fourier transformed k^2-weighted $\chi(k)$ curves of the CS:PVA membrane samples as a function of the type of filler support for: (a,b) unsupported Cu NPs, (c,d) CuAM-4 layered titanosilicate, and (e,f) CuUZAR-S3 layered stannosilicate. Standard patterns for Cu foil (Cu(0)), CuO (Cu(II)) and Cu_2O (Cu(I)) are shown black, light gray and gray, respectively, for comparison.

Influence of water uptake on the thermal stability of the membranes.

Table S2. Main thermogravimetric events in Figure 8b [1].

Membrane[1]	Weight loss at 119 ºC (wt.%)	T_2 (ºC)	WC (wt.%)
CS:PVA	13 ± 4	274	22
5Cu/CS:PVA	13.3	334	13.3
10Cu/CS:PVA	12.1	322	34.8
5CuAM-4/CS:PVA	10.0	272	22.2
10CuAM-4/CS:PVA	9.23	273	23.4
15CuAM-4/CS:PVA	9.88	294	25.8
5CuUZAR-S3/CS:PVA	8.72	298	25.4
10CuUZAR-S3/CS:PVA	7.75	280	24.0
5CuY/CS:PVA	8.84	295	27.3
10CuY/CS:PVA	10.9	287	23.6
5CuMOR/CS:PVA	6.75	275	17.8
10CuMOR/CS:PVA	11.0	281	19.1
5CuBEA/CS:PVA	11.9	254	16.2
10CuBEA/CS:PVA	8.82	246	14.0

[1] The values in this table are the average of three replicas, with an experimental standard deviation within 6%.

Analysis of variance: effects of the preparation variables in membrane properties.

Table S3. ANOVA table for IEC (mmol/g)

**Factor**	**Sum of Squares (SS)**	**Degree of freedom (df)**	**Mean square (MS)**	**F-value**	***p*-value**
Main effects					
A: Type of filler	0.032	5	0.006	0.556	0.742
B: Filler loading	0.029	1	0.029	2.555	0.251
C: Cu content	0.008	1	0.008	0.733	0.482
Interactions:					
A · B	0.021	5	0.004	0.367	0.842
B · C	0.0	1	$2.0 \cdot 10^{-6}$	0.0	0.991
Residuals	0.023	2	0.011		
Total	0.116	19			

Table S4. ANOVA table for conductivity (mS/cm)

**Factor**	**Sum of Squares (SS)**	**Degree of freedom (df)**	**Mean square (MS)**	**F-value**	***p*-value**
Main effects					
A: Type of filler	0.125	5	0.025	0.205	0.933

B: Filler loading	0.071	1	0.071	0.581	0.526
C: Cu content	0.002	1	0.002	0.015	0.914
Interactions					
A · B	0.062	5	0.012	0.102	0.981
B · C	0.098	1	0.098	0.806	0.464
Residuals	0.244	2	0.130		
Total	0.645	19			

Table S5. ANOVA table for WVP (g /mm^2 h kPa)

**Factor**	**Sum of Squares (SS)**	**Degree of freedom (df)**	**Mean square (MS)**	**F-value**	***p*-value**
Main effects					
A: Type of filler	2.364	5	0.473	1.622	0.424
B: Filler loading	0.105	1	0.105	0.361	0.609
C: Cu content	0.156	1	0.156	0.533	0.541
Interactions					
A · B	0.349	5	0.270	0.926	0.593
B · C	0.183	1	0.183	0.629	0.511
Residuals	0.583	2	0.292		
Total	3.905	19			

Model regression fitting as a function of factors and other variable parameters

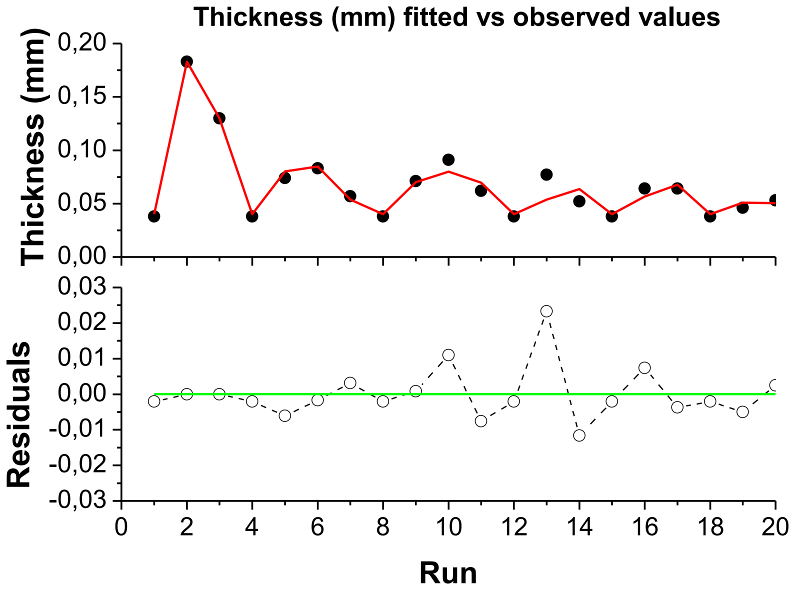

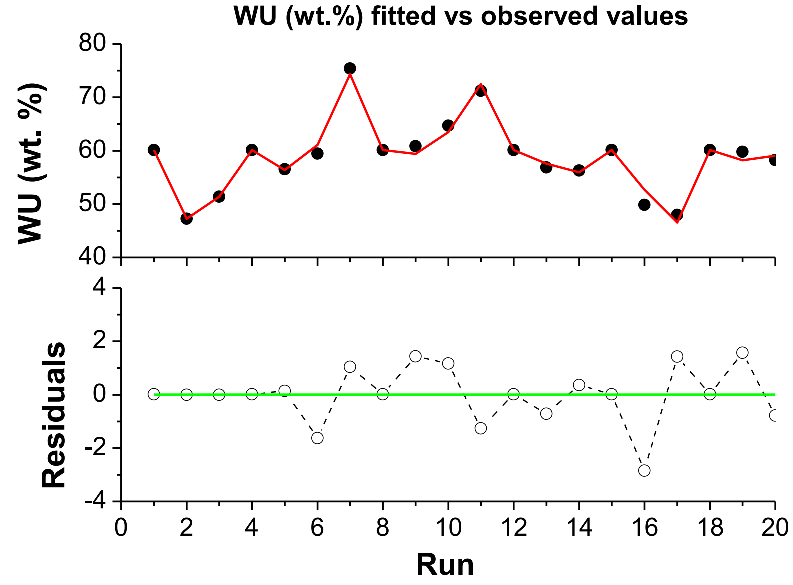

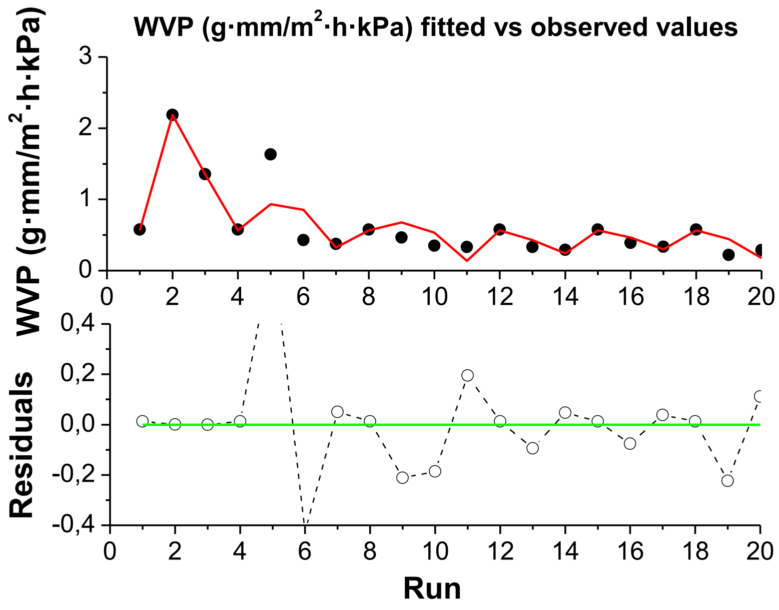

Figure S3. Goodness of fit between observed and fitted values for the thickness, WU, and WVP parameters using the linear multiple regression models.

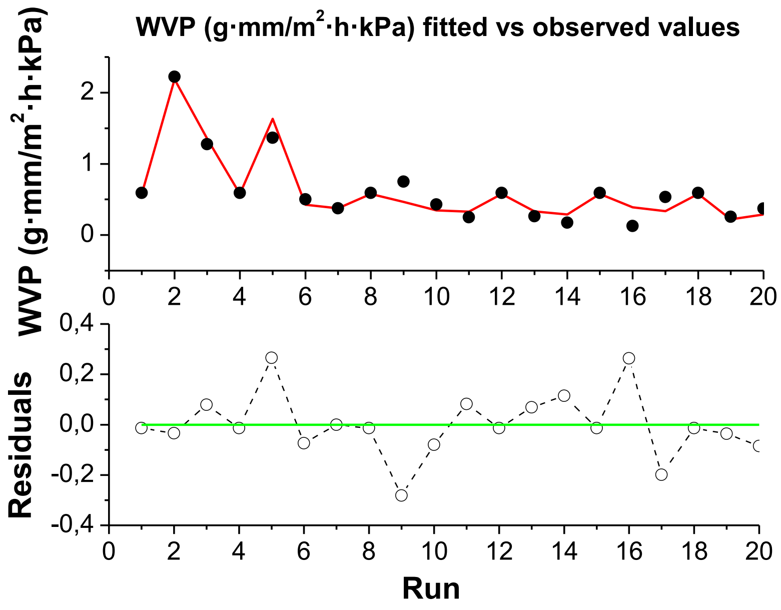

Figure S4. Goodness of fit between observed and fitted values for the WVP parameter using the quadratic multiple regression model.
